# PNT1 Is a C11 Cysteine Peptidase Essential for Replication of the Trypanosome Kinetoplast*

**DOI:** 10.1074/jbc.M116.714972

**Published:** 2016-03-03

**Authors:** Jaspreet S. Grewal, Karen McLuskey, Debanu Das, Elmarie Myburgh, Jonathan Wilkes, Elaine Brown, Leandro Lemgruber, Matthew K. Gould, Richard J. Burchmore, Graham H. Coombs, Achim Schnaufer, Jeremy C. Mottram

**Affiliations:** From the ‡ Wellcome Trust Centre for Molecular Parasitology, Institute of Infection, Immunity and Inflammation, College of Medical, Veterinary and Life Sciences, University of Glasgow, Glasgow G12 8TA, United Kingdom,; the § Department of Biology, Centre for Immunology and Infection, University of York, Wentworth Way, Heslington, York YO10 5DD, United Kingdom,; the ¶ Joint Center for Structural Genomics,; ‖ Stanford Synchrotron Radiation Lightsource, SLAC National Accelerator Laboratory, Menlo Park, California 94025,; the ** Institute of Immunology and Infection Research and Centre for Immunity, Infection, and Evolution, University of Edinburgh, Edinburgh EH9 3FL, United Kingdom,; the ‡‡ Strathclyde Institute of Pharmacy and Biomedical Sciences, University of Strathclyde, Glasgow G4 0RE, United Kingdom

**Keywords:** microscopy, parasite, peptidase, RNA interference (RNAi), Trypanosoma brucei

## Abstract

The structure of a C11 peptidase PmC11 from the gut bacterium, *Parabacteroides merdae*, has recently been determined, enabling the identification and characterization of a C11 orthologue, PNT1, in the parasitic protozoon *Trypanosoma brucei.* A phylogenetic analysis identified PmC11 orthologues in bacteria, archaea, Chromerids, Coccidia, and Kinetoplastida, the latter being the most divergent. A primary sequence alignment of PNT1 with clostripain and PmC11 revealed the position of the characteristic His-Cys catalytic dyad (His^99^ and Cys^136^), and an Asp (Asp^134^) in the potential S_1_ binding site. Immunofluorescence and cryoelectron microscopy revealed that PNT1 localizes to the kinetoplast, an organelle containing the mitochondrial genome of the parasite (kDNA), with an accumulation of the protein at or near the antipodal sites. Depletion of PNT1 by RNAi in the *T. brucei* bloodstream form was lethal both in *in vitro* culture and *in vivo* in mice and the induced population accumulated cells lacking a kinetoplast. In contrast, overexpression of PNT1 led to cells having mislocated kinetoplasts. RNAi depletion of PNT1 in a kDNA independent cell line resulted in kinetoplast loss but was viable, indicating that PNT1 is required exclusively for kinetoplast maintenance. Expression of a recoded wild-type *PNT1* allele, but not of an active site mutant restored parasite viability after induction *in vitro* and *in vivo* confirming that the peptidase activity of PNT1 is essential for parasite survival. These data provide evidence that PNT1 is a cysteine peptidase that is required exclusively for maintenance of the trypanosome kinetoplast.

## Introduction

*Trypanosoma brucei* is a kinetoplastid protozoan parasite and the causative agent of human African trypanosomiasis (sleeping sickness) and nagana in cattle. Sleeping sickness causes widespread human morbidity and death in sub-Saharan Africa. Nagana leads to cattle mortality, lower meat and milk production, and lower calving rate. The parasite has a complex life cycle spanning both the tsetse fly and the mammalian host. The metacyclic trypomastigote is transmitted to the mammalian host by the bite of the tsetse fly. Once inside the human host, these parasites transform into bloodstream form (BSF)^2^ trypomastigotes that divide and multiply in blood and lymph, and which is followed by invasion of the parasites to other organs and the central nervous system. The sleep-wake cycle gets disrupted and if the disease is left untreated, the infected individual enters coma and eventually dies (1). Approximately 60 million people are at risk of being infected worldwide with this disease (WHO fact sheet May 2015). Currently no vaccines are available and the drugs in use are becoming ineffective and are toxic (2, 3). It is therefore imperative that new drug targets are identified against the protozoan parasite.

Cysteine peptidases of parasitic protozoa are associated with important biological processes such as, invasion of the host cells (in case of intracellular parasites) and subsequent pathogenesis (4, 5). Clan CD is comprised of peptidase families that have a protein-fold similar to the caspase family (C14). Clan CD is exemplified by several important cysteine peptidases such as GPI8 (family C13), a component of the GPI-protein transamidase complex (6), metacaspase (family C14B) (7, 8), separase (family C50) (9), and a relatively less characterized family C11 (clostripain) (10). In *T. brucei* the GPI-protein transamidase is required for anchoring proteins to the plasma membrane. Among these proteins the most prominent is variant surface glycoprotein, which forms a monolayer on the parasites surface and functions in the evasion of the immune system of the host (6). *T. brucei* has five metacaspases, including MCA4, which is a pseudopeptidase and virulence factor (11), and MCA2, which is a calcium-dependent enzyme associated with RAB11 positive endosomes and does not require processing for activation (12, 13). Another *T. brucei* cysteine peptidase, separase, functions in segregation of mini-chromosomes and proper mitotic assembly (14).

Recently, the first crystal structure of a family C11 peptidase, PmC11, was determined from the gut bacterium *Parabacteroides merdae* (15). This structure facilitated the identification of an important *T. brucei* protein, PNT1 (Puf Nine Target 1) as a C11 orthologue and a potential cysteine peptidase. The *PNT1* transcript was previously described, in the insect procyclic form of the parasite, as the target of the RNA-binding protein PUF9 (16), with PNT1 localizing to the kinetoplast, a unique organelle containing the mitochondrial DNA of the organism. The kinetoplast DNA (kDNA) of *T. brucei* is composed of a few dozen maxicircles (23 kb) and several thousand minicircles (∼1 kb) (17). The maxicircles encode essential mitochondrial proteins including the respiratory chain complex subunits. The minicircles encode guide-RNAs that function in editing the RNA encoded by the maxicircles. The division of the mitochondrial DNA is coordinated with cytokinesis (18) and the presence of essential genes on the kinetoplast makes it imperative for each of the *T. brucei* progeny cells to inherit a kinetoplast (19). Related species, *Trypanosoma equiperdum* and *Trypanosoma evansi*, do not have a fully functional kinetoplast and are, therefore, known as dyskinetoplastic (20). RNAi studies on kinetoplast regulatory genes have shown that loss of the kinetoplast in *T. brucei* is lethal; hence, the organelle is considered an important drug target (19, 21). Our functional analysis suggests that PNT1 is a peptidase that plays an essential role in the maintenance of the kinetoplast, and that a catalytically active PNT1 is required for the survival of *T. brucei*. This work also demonstrates that depletion of PNT1 leads to the generation of an akinetoplastic cell line, whereas overexpression leads to formation of mislocated kinetoplasts thus, inhibitors of PNT1 could be of interest for drug discovery programs.

## Experimental Procedures

### 

#### 

##### Phylogenetic Analysis

The peptidase C11 hidden Markov model (HMM) profile (ACC PF03415.9) was obtained from Pfam. The C11 peptidases are members of clan CD, a group characterized by a doublet (H(G/A)) and triplet (D(A/S)C) motif accounting for the catalytic histidine/cysteine dyad (22). The UniProtKB FASTA set (04/29/2015) was downloaded from the UniProt site. This sequence set was searched for peptides containing domains consistent with the model (conformant proteins) by use of the HMM search program of HMMER3 using default parameters. The hits (full sequence *E* value <0.0001) were filtered as follows. Sequences from prokaryotic and archaeal species were excluded; eukaryotic alignments were examined and only those with at least a minimum alignment to the cysteine containing motif (D(A/S)C) retained. Full peptide sequences for the identified eukaryotic sequences were subsequently retrieved from UniprotKB; and a representative sample of bacterial and archaeal C11 peptidase sequences were obtained from the MEROPS database and added to the sequence set.

The meta-method for assembling multiple sequence alignments was performed on the sequence set using the M-Coffee (23) mode of T-Coffee (24). The output alignment (color coded for alignment quality) was examined visually to determine the domain containing the doublet (H(G/A)) and triplet (D(A/S)C). The alignment was trimmed to this domain, and an HMM profile generated (C11_euk), using the HMMER3 program hmmbuild. This HMM profile was used to search proteome sets of a variety of eukaryotic genomes for C11_euk conformant sequences. The domain sequences were extracted, the sequence for metacaspase TbMCA2 (*T. brucei* Tb927.6.940, MEROPS family C14, also in clan CD) was added as an outgroup and a multiple sequence alignment was generated as above. Low quality (<4) and low occupancy (<30%) sites were removed from the alignment and a neighbor-joining tree generated from the refined alignment (SplitsTree4 (25)).

##### Trypanosome Cell Culture

*T. brucei* 2T1 BSF cells were maintained in HMI-9 medium supplemented with 10% FBS (low TET, GIBCO), 100 unit ml^−1^ of penicillin, 100 μg ml^−1^ of streptomycin. The parasites were transfected as described (26) and clones were obtained by limiting dilution. Appropriate concentrations of antibiotics were used for the selection and maintenance of the parasites: 2 μg ml^−1^ of puromycin, 2.5 μg ml^−1^ of phleomycin, 5 μg ml^−1^ of hygromycin B, and 10 μg ml^−1^ of blasticidin (all from InvivoGen). RNAi was induced using 1 μg ml^−1^ of tetracycline. Cells were grown in 24-well plates in triplicate for growth curve experiments.

##### Generation of the RNAi, Recoded and Myc-tagged PNT1 Constructs

A *PNT1* RNAi construct (pTL232) was generated using pGL2084, a gateway compatible version of pRPa^SLi^ (27, 28). The oligonucleotides used for the generation of sense and antisense *PNT1* inserts for pTL232 were: OL4052, GGGGACAAGTTTGTACAAAAAAGCAGGCTCGTCAGGACATGCTTGAAGA, and OL4053, GGGGACCACTTTGTACAAGAAAGCTGGGTTTTGCAATTCTTTCTGTGCG). pTL232 was digested with AscI and the stem loop cassette was transfected in the bloodstream form of *T. brucei* 2T1. A recoded gene (encoding mRNA that is refractory to RNAi) of *PNT1* was designed in CLC genomics workbench (CLCbio) using the codon usage table of *T. brucei* and subsequently synthesized by GeneArt (Thermo Fisher Scientific). The recoded *PNT1* gene was cloned in-frame with a C-terminal HA tag in pGL2243 (29), which integrates the gene of interest at the tubulin locus for constitutive expression. Site-directed mutagenesis was carried out on the recoded *PNT1* to generate an active site recoded mutant *PNT1HA^C136A^*. The mutations were carried out using the QuikChange Site-directed Mutagenesis kit (Stratagene) as per the manufacturer's protocol. The identifiers of the *PNT1HA* recoded and the mutated constructs were pGL2278 and pGL2320, respectively. The oligonucleotides used to generate the mutated recoded *PNT1* were OL4280, CGATGGTTTTTGATAGTAGTTTCATGGCGAGCCTGG, and OL4281, CCAGGCTCGCCATGAAACTACTATCAAAAACCATCG. *PNT1* was cloned in-frame in a tetracycline inducible C-Myc_6_ tag vector (pGL2220) (27) for generating a *PNT1Myc* cell line.

##### Transfection of the Parasites

1 × 10^7^ log phase BSF cells were suspended in 100 μl of ice-cold Amaxa Human T-Cell solution (Amaxa, Koeln, Germany) and transfection was carried out according to the manufacturer's protocol. Briefly, 10 μg of linearized DNA plasmid was added to the cells and transfected with the Amaxa Human Nucleofector II, using program X-001. The cells were transferred to pre-warmed HMI 11 medium and plated out in 24-well plates at 1:20 and 1:40 dilutions. The selection antibiotic was added after 6 h of transfection and cells were incubated at 37 °C. Transfectants were selected after 1 week. F_1_F_O_-ATPase subunit γL262P mutants (20) were generated in the *PNT1* RNAi cell line using gene replacement constructs.^3^ Briefly, two rounds of transfections were carried out to replace both the endogenous subunit γ alleles with γL262P mutant alleles. The cell lines were tested for kDNA independence by treating with 10 nm ethidium bromide for four passages, which resulted in generation of viable akinetoplastic cells.

##### Analysis of Trypanosome Growth after Induction of RNAi

The *PNT1* RNAi cell lines were seeded in triplicate at 1 × 10^5^ cells per well in 24-well plates (HMI 11 hygromycin-phleomycin media) in the presence and absence of tetracycline (1 μg ml^−1^). The cell numbers were counted using a hemocytometer every 24 h and cell seeded at 1 × 10^5^ cells for the subsequent time point. Infections in mice were carried out by initially inoculating a donor mouse with 1 × 10^5^ parasites and monitoring blood parasitemia over 72 h. Four experimental mice were infected with 4 × 10^4^ parasites harvested from the donor mouse. Doxycycline was added to the drinking water of two of the mice on the day of inoculation. Parasitemia in the blood of the mice was calculated daily using a hemocytometer after drawing the blood by tail venipuncture and diluting the blood in 0.83% ammonium chloride. Cumulative growth curves were plotted for the *in vivo* and *in vitro* cell counts using Prism6 (GraphPad software). The experiments were done in triplicate for *in vitro* and in duplicate for *in vivo* experiments.

##### Real Time-PCR

2 × 10^5^ BSF cells were grown for 24 h in the presence and absence of tetracycline, harvested, and RNA was prepared using the RNeasy kit (Qiagen). First strand cDNA was prepared using 4 μg of RNA for each sample (Invitrogen Superscript: first strand synthesis kit). The cDNA was treated with RNase H (Invitrogen) to degrade the template RNA. PCR was performed using SYBR Green PCR master mix (Applied Biosystems) and *GPI8* was used as an endogenous control. Oligonucleotides OL4119, CACTTGGGTGGGCTCTCTTTC, and OL4120, CAGCGGCTTCGACAATGG, were used for amplifying *PNT1*, and OL2272, CGAAGCGCATTTGGATAGC, and OL2273, AGCGCGTGATGACAGTGAAG, to amplify *GPI8*. The quantitative PCR was run and analyzed on a 7300 Real-time PCR system (Applied Biosystems).

##### SDS-PAGE and Western Blotting of T. brucei Cell Lysates

1 × 10^7^ BSF parasites were lysed in sample buffer (NuPAGE sample buffer) and analyzed on a 4–12% NuPAGE Novex BisTris gel using MES buffer. Subsequently, the samples were electroblotted on nitrocellulose membrane (GE Healthcare) and probed with the respective antibodies. The *PNT1HA* cell line was probed with anti-HA antibody (1:4000, Roche Applied Science). The *PNT1Myc* cell line was probed with anti-Myc antibody (1:2000, Millipore). Anti-EF1α antibody (1:5000, Millipore) was used for the loading controls.

##### Immunofluorescence

DAPI staining was carried out using 10^6^ cells from exponential phase cultures. The cells were washed with vPBS (22, 28), added to a 12-well glass slide (Thermo Fisher) pre-coated in poly-l-lysine (0.1% (w/v) in H_2_O, Sigma), and allowed to adhere for 4 min. Cells were fixed with of 4% paraformaldehyde for 5 min. The wells were washed twice with PBS (pH 7.4) and excess paraformaldehyde was quenched with 100 mm glycine for 10 min. The cells were permeabilized with 0.1% Triton X-100 for 10 min. The wells were washed three times with PBS and overlaid with a drop of SlowFade Gold Antifade Reagent with DAPI (Invitrogen). Coverslips were added and the slides were kept at 4 °C until required. Staining of PNT1HA was performed using 1 × 10^7^ cells from an exponential phase culture. The cells were resuspended in 200 μl of PBS followed by addition of 300 μl of 4% paraformaldehyde to fix overnight at 4 °C. The cells were washed with PBS and resuspended in 100 mm glycine for 15 min. The cells were then centrifuged at 600 × *g* for 10 min and resuspended in 0.1% Triton X-100 (Sigma) for 5 min. Cells were washed twice with PBS and incubated on ice for 1 h in 1% BSA (in PBS). The cells were centrifuged for 600 × *g* for 10 min and incubated with anti-HA antibody (1:200, rat monoclonal, Roche) for 1 h on ice. After washing with 0.1% BSA (PBS) cells were resuspended in anti-rat Alexa 488 (1:2000, Molecular Probes) and incubated in the dark on ice for 1 h. The cells were washed with 0.1% BSA (PBS) before 0.5 μl of DAPI (100 μg/ml stock) was added to the cells and incubated for 5 min. The cells were washed once with 0.1% BSA (PBS) and once with PBS. The final cell pellet was resuspended in final volume of PBS (30–50 μl) and stored at 4 °C until ready to image. The staining of the *PNT1Myc* cell line was also carried out as described above using an anti-Myc antibody conjugated to Alexa Fluor 488 (Millipore) at 1:500 dilution. Stained cells were spread on a slide and overlaid with a square coverslip. The images were acquired on a DeltaVision Core microscope using excitation wavelength of 405–489 nm and images were analyzed and processed in Adobe Photoshop and FIJI.

##### Transmission Electron Microscopy

Cells were washed in PBS, fixed with 2.5% glutaraldehyde and 4% paraformaldehyde in 0.1 m sodium cacodylate buffer (pH 7.2), and post-fixed with 1% osmium tetroxide and 2.5% potassium ferrocyanide in 0.1 m sodium cacodylate buffer (pH 7.3) for 45 min in the dark. Samples were *en bloc* stained with 2% aqueous uranyl acetate, dehydrated in a series of acetone solutions (30, 50, 70, 90, and 100%), and finally embedded in Epon resin. Collected ultrathin sections were stained with lead citrate prior to observation in a Tecnai T20 transmission electron microscope (FEI, Netherlands). All images were analyzed and processed in Adobe Photoshop and FIJI (30). Acquired images were processed and the intensity plot profile was acquired for kinetoplast density analysis. Statistical analyses were performed using Prism6, with one-way analysis of variance analysis. Immunogold labeling of cells was done by fixing cells overnight in 4% paraformaldehyde in phosphate buffer, and dehydrating in ascending ethanol solutions. The material was progressively infiltrated with LR White resin, and polymerization was carried out in gelatin capsules under ultraviolet light. Formvar-coated nickel grids with ultrathin sections were incubated with blocking solution (3% BSA, 0.02% Tween 20, in phosphate buffer, pH 8) for 1 h. The grids were then incubated with the primary antibody anti-HA (1:200 rat monoclonal, Roche) diluted in blocking buffer for 1 h followed by several washes in blocking buffer. The grids were then incubated with protein A conjugated to immunogold (Aurion, Netherlands) for 1 h. After several washes with blocking buffer and phosphate buffer, the grids were stained with uranyl acetate and lead citrate prior to observation in a Tecnai T20 transmission electron microscope (FEI, Netherlands).

## Results

### 

#### 

##### Phylogeny of the C11 Cysteine Peptidase Family

**FIGURE 1.**
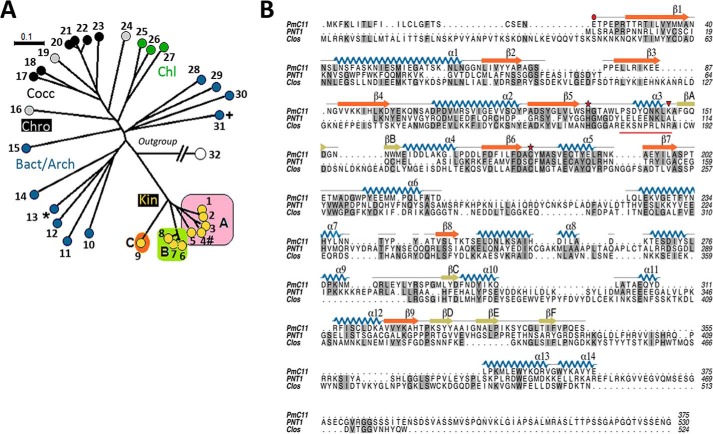
**Phylogenetic analysis of the clan CD C11 peptidase family.** *A,* unrooted neighbor joining tree of the C11 family from bacteria and archaeal species (*Bact*/*Arch*), Coccidia (*Cocc*), Chromerida (*Chro*), Chlorophyta (*Chl*), and Kinetoplastids (*Kin*; *A, Trypanosoma; B, Leishmania*/*Endotrypanum*/*Crithidia; C, Bodonida*). Clostripain (+)Uniprot ID P09870; PmC11(*)Uniprot ID, A7A9N3; and PNT1 (#)Uniprot ID, Q385B4 are marked. Key: *1,* K2PE90 *Trypanosoma cruzi. 2,* A0A061IWY5 *Trypanosoma rangeli. 3,* F9W8X5 *Trypanosoma congolense. 4,* Tb927.11.6550 *T. brucei. 5,* G0UBL1 *Trypanosoma vivax. 6,* Q4QH06 *Leishmania major. 7,* AOFS01001278.1 2 2 2134 *Endotrypanum monterogeii. 8,* CfaC1 13 0800 *Crithidia fasciculata. 9,* BS39455 *B. saltans. 10*, EQ973215 *Bacteroides fragilis. 11*, GL883824 *Paraprevotella xylaniphila. 12*, AHHG01000029 *Elizabethkingia anophelis. 13*, P09870 *C. histolyticum. 14*, ADLW01000010 *Dysgonomonas mossii. 15*, AE010299 *Methanosarcina acetivorans. 16*, SNAP00000001341 *Eimeria tenella. 17*, B6ABJ5 *Cryptosporidium muris. 18*, MER037012 *Cryptosporidium parvum. 19*, Cvel 15101 *Chromera velia. 20*, A0A086LCT5 *Toxoplasma gondii. 21*, V4ZRY6 *T. gondii. 22*, F0VJH3 *Neospora caninum. 23*, U6G6U0 *Eimeria praecox. 24*, MER494436 *Vitrella brassicaformis. 25*, D8TW66 *Volvox carteri. 26*, A8I9P1 *Chlamydomonas reinhardtii. 27*, A0A0D2MVH2 *Monoraphidium neglectum. 28*, NC 018015 *Thermococcus* sp. CL1. *29*, ABYK01000024 *Arthrospira maxima. 30*, DS990529 *Aciduliprofundum boonei. 31*, A7A9N3 *P. merdae. 32*, AJ437303 *T. brucei* MCA2 (outgroup). *B,* primary amino acid sequence alignment of PmC11 (from *P. merdae*), PNT1 (from *T. brucei*), and clostripain (from *C. histolyticum*) with identical residues highlighted in *gray shading*. The assigned secondary structure from the crystal structure of PmC11 (15) is mapped onto its sequence with the position of the PmC11 catalytic dyad and autocatalytic cleavage site (Lys^147^) highlighted by a *red star* and a *red triangle*, respectively. Connecting loops are colored *gray*/*black*, the main β-sheet is in *orange*, with other strands in *olive*, α-helices in *blue*, and the nonapeptide linker of clostripain *underlined in red*. Sequences around the catalytic site of clostripain, PmC11, and PNT1 are aligned where identical residues are highlighted in *gray*.

To determine the phylogenetic distribution of C11 proteins, the UniProt database was queried with a bespoke C11 HMM domain profile. This analysis identified clostripain-like proteins in a wide range of bacterial and archaeal species and in a limited range of eukaryotic species, which could be divided into distinct clades (branches) (Fig. 1*A*). One clade, containing a subset of species from *Chromerida, Coccidia*, and *Chlorophyta*, is monophyletic with the archaeal and bacterial species, as exemplified by *P. merdae* PmC11 (15) and *Clostridium histolyticum* clostripain, and suggests a lateral gene transfer event from a non-eukaryotic species to a plastid-containing organism ancestral to these phyla (31). The sparse distribution of the C11 domain is indicative of frequent loss from genomes in successive rounds of evolution, as there is no evidence of C11 proteins among species of *Babesia* or *Plasmodium,* whereas C11 proteins are found in other apicomplexan genera including *Toxoplasma*, *Eimeria*, and *Neospora*. A second clade (kin) is present in kinetoplastids and is atypical in comparison to other eukaryotic species and bacterial/archaeal domains. The C11 domain is found in *T. brucei* (PNT1), *Leishmania*, and *Bodo saltans*, but not in *Phytomonas*, *Leptomonas*, or *Paratrypanosoma*, indicating secondary loss of the *PNT1* gene in kinetoplastid groups.

##### Active PNT1 Is Essential for T. brucei Viability

PNT1 is the ortholog of PmC11 in *T. brucei* (Fig. 1*A*). A primary sequence alignment of PmC11, clostripain, and PNT1 (Fig. 1*B*) led to the identification of the conserved His-Cys catalytic dyad (His^99^ and Cys^136^, respectively). PmC11 is somewhat shorter than the other two proteins but large sections of all three overlay well, covering most of the PmC11 residues with particularly good alignment around the active site. This suggests that the secondary structural elements identified in PmC11 will form the core structural elements of clostripain and PNT1.

**FIGURE 2.**
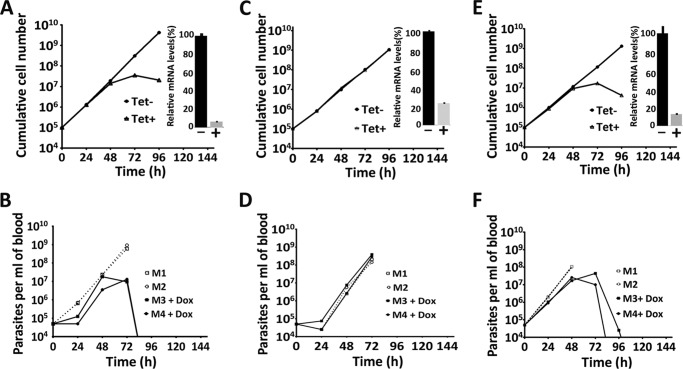
**Growth of *PNT1* RNAi cell lines *in vitro* and *in vivo*.** *A* and *B*, *PNT1* RNAi cell line. *C and D*, *PNT1* RNAi cell line expressing recoded PNT1HA. *E* and *F*, *PNT1* RNAi cell line expressing recoded PNT1HA^C136S^. *A, C*, and *E,* representative growth curves show cumulative cell counts after induction of RNAi with tetracycline *in vitro* (mean of three independent experiments) (*inset*, *PNT1* mRNA depletion monitored by qRT-PCR). *B, D*, and *F*, 4 mice were inoculated intraperitoneally with 4 × 10^4^ parasites and RNAi was induced with doxycycline in 2 mice at the time of inoculation. Uninduced mice were culled when the parasitemia levels were higher than 10^8^ cells ml^−1^. The parasitemia in blood samples were plotted over time.

Attempts to obtain soluble recombinant PNT1, for testing protease activity *in vitro*, were unsuccessful. However, it was possible to investigate peptidase activity and its importance in parasite survival indirectly. Initially, a *PNT1* RNAi line was generated in the BSF of *T. brucei*, where induction by tetracycline resulted in loss of cell viability in the *in vitro* culture (Fig. 2*A*). PNT1 was also observed to be essential *in vivo* with doxycycline induction leading to loss of detectable parasitemia in mice after 96 h (Fig. 2*B*).

**FIGURE 3.**
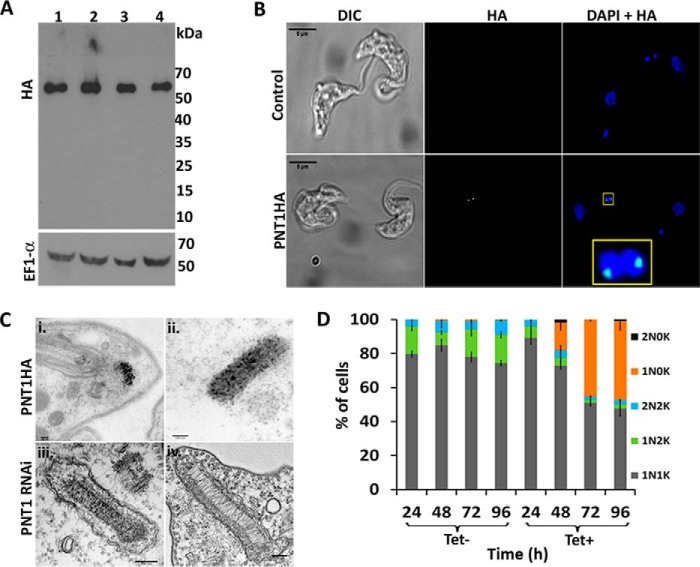
**Phenotype analysis of *PNT1* RNAi, *PNT1HA*, and *PNT1Myc* cell lines.** *A,* expression of recoded PNT1HA in the *PNT1* RNAi cell line. Lysates of *PNT1* RNAi cells expressing recoded PNT1HA (*lanes 1* and *2*) or recoded PNT1HA^C136S^ (*lanes 3* and *4*) before (*lanes 1* and *3*) and after (*lanes 2* and *4*) tetracycline induction were immunoblotted and probed with anti-HA antibody. EF1α was used as loading control. *B*, immunofluorescence of PNT1HA expressed in tetracycline induced *PNT1* RNAi cell line (*lower panel*) shows localization within or near antipodal sites. Uninduced *PNT1* RNAi cell lines (*upper panel*) were used as a control. The cells were stained with anti-HA antibody (*green*) and DAPI (*blue*). A stained, nearly duplicated kinetoplast (*yellow box*) is shown in magnified view. Scale bar, 5 μm. *C, i* and *ii*, immunogold labeling of *PNT1HA* cell line using anti-HA antibody after 72 h of RNAi induction. *Scale bar,* 100 nm. *iii* and *iv,* electron micrographs of *PNT1* RNAi uninduced (*iii*) and 72 h tetracycline-induced (*iv*) cells (*scale bar*, 150 nm). *D, PNT1* RNAi cells induced with tetracycline (*Tet*+) were stained with DAPI and the number of nuclei (*N*) and kinetoplasts (*K*) per cell was counted (in 200 cells) at the indicated time points and plotted as a percentage of the total. Each histogram represents mean ± S.D. (*n* = 3).

To test whether catalytically active PNT1 is required for the survival of the parasites, a recoded version of *PNT1*, containing a C-terminal HA tag (*PNT1HA*, which is refractory to RNAi) was constitutively expressed in the *PNT1* RNAi line. Expression of the PNT1HA recoded protein compensated for the loss of endogenous PNT1 and restored viability of tetracycline induced parasites both *in vitro* culture (Fig. 2*C*) and *in vivo* (Fig. 2*D*). A cell line in which the recoded *PNT1HA* had the predicted active site cysteine mutated to serine PNT1HA^C136S^ was not viable after induction *in vitro* (Fig. 2*E*) and *in vivo* (Fig. 2*F*). Quantitative RT-PCR confirmed the depletion of *PNT1* mRNA in each of the cell lines (*insets* in Fig. 2, *A*, *C,* and *E*) and Western blotting confirmed that PNT1HA^C136S^ was expressed at a similar level to PNT1HA (Fig. 3*A*). Another interesting observation from the Western blots was the absence of processed fragments of recoded PNT1HA suggesting that PNT1 might not require processing for activity. This is in contrast to PmC11, which requires intramolecular processing for full activation (15). This observation is further supported by the primary sequence alignment of PNT1 with PmC11 and clostripain, where the cleavage site Lys (Lys^147^) in PmC11 aligns with a reported cleavage site Arg (Arg^190^) in clostripain (32), whereas in PNT1 there is an Ala at the equivalent position (Fig. 1*B*). Additionally, phylogenetic analysis (Fig. 1*A*) shows that trypanosomatid C11 orthologues are quite divergent from bacterial and archaea C11 family members. These results suggest that PNT1 is different from PmC11 in terms of activation and that the peptidase activity of PNT1 is essential for viability of *T. brucei*.

##### PNT1 Localizes on the Kinetoplast

**FIGURE 4.**
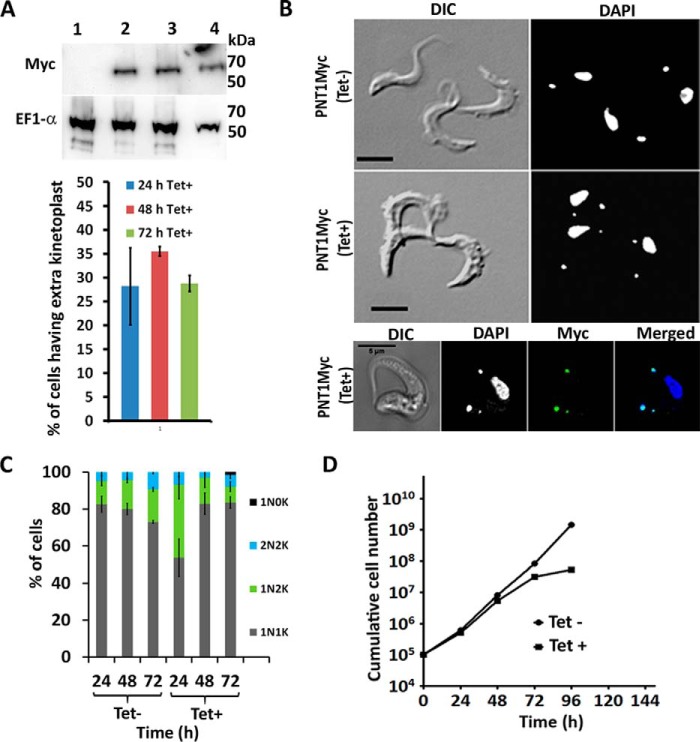
**Overexpression of PNT1.** *A, upper panel*, Western blot showing expression of PNT1Myc using anti-Myc antibody at different time points after tetracycline induction (*lanes 2–4* represent induced samples after 24, 48, and 72 h, respectively). A 24-h uninduced PNT1Myc sample in *lane 1* served as negative control. EF1α was used as the loading control. *Lower panel*, percentage of cells having anterior kinetoplasts after tetracycline induction at different time points (quantitation from 200 cells, histogram represents mean ± S.D. (*n* = 3)). *B*, representative images showing the DAPI-stained, *PNT1Myc*-tagged cell line before (*top panel*) and after (*middle panel*) induction with tetracycline for 24 h. *Bottom panel*, staining of the *PNT1Myc* cell line with anti-Myc antibody conjugated to Alexa Fluor 488 (*green*). *Scale bar*, 5 μm. *C*, counts of DAPI-stained kinetoplast (*K*) and nuclei (*N*) of the *PNT1Myc* cell line showing a higher percentage of 1N2K cells at 24 h after induction. The number of 1N2K cells fall after 48 and 72 h (*n* = 3, mean ± S.D.). *D*, cumulative growth curve of *PNT1Myc* cell line showing reduced cell viability after PNT1 overexpression.

PNT1 localizes to the kinetoplast in BSF parasites and was enriched either within or near the antipodal sites, which are protein assemblies located on opposite sides of the kDNA disc involved in minicircle replication (33, 34). This localization was more prominent in dividing kinetoplasts (Fig. 3*B*). Immunogold labeling of the *PNT1HA* cell line confirmed that PNT1HA is located on the kinetoplast (Fig. 3*C, i* and *ii*)). The status of the kinetoplast in PNT1-depleted cells was investigated using TEM, which showed a lower electron density of the kinetoplast in RNAi-induced cells compared with uninduced cells (Fig. 3, *iii* and *iv*). This indicates that PNT1 depletion results in a reduction of material within the kinetoplast. It has been demonstrated that RNAi-mediated depletion of proteins closely associated with the kinetoplast can cause the loss of kDNA (35, 36). To observe the effects of down-regulating *PNT1* on the number of nuclei and kinetoplasts, the *PNT1* RNAi line was stained with DAPI at different time points after induction. Depletion of *PNT1* by RNAi resulted in an increased number of cells lacking a kinetoplast (akinetoplastic, 1N0K) after 2 days (Fig. 3*D*). The number of 1N0K cells increased from 16% on day 2 to 46% on day 4, after RNAi induction. This increase in akinetoplastic cells suggests that PNT1 is involved in the maintenance of the kinetoplast in *T. brucei*. Interestingly, inducible overexpression of a Myc-tagged PNT1 (PNT1Myc) leads to formation of an additional kinetoplast anterior to the nucleus, a phenotype similar to that observed in the procyclic form of *T. brucei* (16). In addition, 36% of the induced *PNT1Myc* cell line had a mislocated kinetoplast after 48 h of induction (Fig. 4, *A* and *B*). PNT1 was also localized on these incorrectly positioned kinetoplasts (Fig. 4*B, lower panel*).

Overexpression of PNT1 led to an increase of 1N2K cells from 12 to 39% after 24 h of induction, compared with 12% observed in uninduced cells (Fig. 4*C*). However, the number of 1N2K cells in the induced culture decreased to 14 and 8% at 48- and 72-h time points, respectively. The *T. brucei* cell cycle is a well coordinated event in which the kinetoplast is placed posterior to the nucleus during cytokinesis. In cells overexpressing PNT1, mislocated kinetoplasts found anterior to the nucleus were observed in cells at all stages of the cell cycle. The decrease in the number of kinetoplasts might be due to continuous cytokinesis of cells having mislocated kinetoplasts. PNT1 overexpression by tetracycline induction also leads to loss of cell viability in BSF *T. brucei* (Fig. 4*D*). Importantly, cell death is observed when PNT1 is either down-regulated or overexpressed, indicating that an optimal amount of PNT1 peptidase activity is essential for viability of the parasite.

##### PNT1 Is Required Exclusively for Maintenance of the Kinetoplast

**FIGURE 5.**
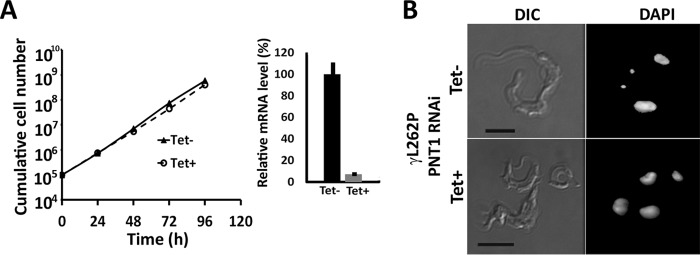
**PNT1 is exclusively required for kinetoplast function.** *A, left panel*, cumulative growth curve of *PNT1*γ*L262P* RNAi line over time ± tetracycline (*n* = 3). *Right panel*, the mRNA levels of *PNT1* transcript were evaluated in the induced and uninduced *PNT1*γ*L262P* RNAi cell line using quantitative RT-PCR. Each histogram represents the mean ± S.D. (*n* = 3). *B,* loss of kinetoplast in the *PNT1*γ*L262P* RNAi cell line after addition of tetracycline to down-regulate *PNT1*. The cells were grown for 120 h ± tetracycline and stained with DAPI. *Scale bar*, 5 μm.

A kinetoplast-independent *PNT1* RNAi cell line was generated to investigate whether PNT1 is required exclusively for kinetoplast maintenance or whether it has other essential mitochondrial functions. It has recently been demonstrated that an L262P mutation in the F1γ subunit of the F_1_F_0_-ATPase complex allows BSF *T. brucei* to survive loss of the kinetoplast (20). The *PNT1* RNAi line was modified to replace both of the wild-type γ alleles with L262P mutant alleles. The *PNT1* RNAi was then induced with tetracycline. No growth defect was observed after induction (Fig. 5*A*, *left panel*) despite greater than 90% depletion of the *PNT1* transcript (Fig. 5*A*, *right panel*). Additionally, depletion of PNT1 resulted in a 100% akinetoplastic cell population after 4 days of induction. Thus, PNT1 is redundant in the absence of a requirement for the kinetoplast for cell viability (Fig. 5*B*). Taken together, these data indicate that PNT1 is exclusively required for kinetoplast maintenance and becomes redundant in the absence of a cellular requirement for the organelle.

## Discussion

The structure of a bacterial C11 protein (PmC11) (15) allowed the catalytic dyad of the PNT1 orthologue to be identified and probed. Despite being larger than PmC11, a basic primary sequence alignment suggests that the secondary structural elements identified in PmC11 will form the core structural elements of PNT1 (Fig. 1*B*). Our data and others (16) indicate that PNT1 is present at the ends of the kinetoplast, which could be the antipodal sites where newly replicated minicircles reattach to the network for repairing discontinuities during replication (such as gap filling and ligation) (37, 38). Several proteins have been identified at the antipodal sites of the kinetoplast including helicases (33, 39), a minicircle origin binding protein p38 (40), DNA polymerase β (41), and a DNA primase PRI1 (42), among others. Depletion of p38 (40), PIF8 (33), and PRI1 (42) also result in loss of kDNA, a similar phenotype to PNT1 depletion; however, there is no evidence to suggest that these proteins require proteolytic processing, whereas it is the peptidase activity of PNT1 that is essential. To date, only one other protease, HslVU (43), has been found to regulate kDNA maintenance in *T. brucei*. Unlike PNT1, down-regulation of HslVU leads to over-replication of minicircles and formation of large kDNA networks. Moreover, HslVU localizes throughout the mitochondrion, suggesting that HslVU could have other roles in the organelle, whereas PNT1 is required exclusively for kDNA maintenance. The appearance of an additional kinetoplast in an abnormal position observed after PNT1 overexpression is reminiscent of the ”ancillary“ kinetoplasts reported after ablation of, DNA polymerase IC (44) or the acyl carrier protein (45), and of those observed in candidate gametes of *T. brucei* (46). Phylogenetic analysis of PNT1 orthologues suggests that kinetoplastid *PNT1* genes are very divergent from orthologues of other organisms. The sequence alignment of PNT1 with PmC11 revealed the presence of conserved structural elements, but also showed novel elements indicating that they have evolved to perform some kinetoplastid-specific function. Not all kinetoplastids have PNT1; *Phytomona*s and *Leptomonas* appear to lack a PmC11 orthologue, which might indicate that there are alternative regulatory mechanisms for kDNA replication in these organisms. It is also interesting to note that *T. evansi* and *T. equiperdum* have a *PNT1* gene, even though these organisms are dyskinetoplastic (47). It is possible that PNT1 is required for maintenance of minicircles in these organisms, although there are no data currently to support this hypothesis. Further work on the identification of PNT1 substrates would provide exciting new insights into the mechanism of kinetoplast replication and segregation in *T. brucei* as well as guiding inhibitor and possibly drug discovery.

## Author Contributions

J. S. G., K. M., D. D., and J. C. M. designed the research; J. S. G., K. M., E. M., J. W., E. B., and L. L. performed the research; J. S. G., K. M., E. M., J. W., L. L., R. B., G. H. C., A. S., and J. C. M. analyzed the data; and J. S. G., K. M., G. H. C., and J. C. M. wrote the paper.

## Footnotes

3 M. Gould and A. Schnaufer, unpublished data.

## Footnotes

2 The abbreviations used are:

BSF: bloodstream form
kDNA: kinetoplast DNA
HMM: hidden Markov model
BisTris: 2-[bis(2-hydroxyethyl)amino]-2-(hydroxymethyl)propane-1,3-diol.
